# Suture tape augmentation, a novel application of synthetic materials in anterior cruciate ligament reconstruction: A systematic review

**DOI:** 10.3389/fbioe.2022.1065314

**Published:** 2023-01-03

**Authors:** Tong Zheng, Yanwei Cao, Guanyang Song, Yue Li, Zhijun Zhang, Zheng Feng, Hui Zhang

**Affiliations:** Sports Medicine Department, Beijing Jishuitan Hospital, Beijing, China

**Keywords:** anterior cruciate ligament, suture tape, synthetic materials, augmentation, outcomes

## Abstract

**Objective:** Suture tape (ST) is a common synthetic material in the repairing surgery of soft tissue. Recently, ST augmentation (STA) technique has been described as a novel way to improve the mechanical property of grafts in the anterior cruciate ligament (ACL) reconstruction (ACLR). However, the clinical outcomes of ACLR using ST-augmented grafts have not been clarified. This systematic review aimed to summarize the specific technique of STA and evaluate the clinical outcomes after ACLR with STA.

**Methods:** A electronic search of PubMed and Embase databases with a manual search of Google Scholar was performed to identify studies that reported the clinical outcomes of ACLR with STA. Each included study was abstracted regarding the study features, patient data, surgical information, and outcome measures.

**Results:** Nine studies were included, representing 314 knees in 314 patients undergoing ACLR with STA. Technically, ST was fixed independently from grafts in six studies and along with grafts in two studies. Most studies applied an equal or slightly less tension on ST than ACL graft. Clinically, significant improvements were found in the Lysholm, IKDC, and KOOS scores after a mean follow-up of 16.7 months. Physical examinations of 220 patients showed significant restoration of knee stability at the final follow-up. 59 of 80 (73.8%) patients returned to preinjury sports level at a minimum 2 year follow-up. Six of 266 (2.3%) patients had a graft failure during the first 2 years postoperatively. The use of ST was significantly associated with better Tegner scores and a trend toward significantly higher rates of return to sport compared to standard ACLR. No significant difference was found in most subjective scores, knee laxity, and graft failures between ACLR with or without STA.

**Conclusion:** ACLR with STA achieved overall favorable clinical outcomes. Patients using ST-augmented grafts were seemingly associated with better sports performance compared to standard ACLR. But ACLR with STA was not superior to ACLR alone in most functional scores, knee stability measures, and graft failure rates. A tension equal to or slightly less than the ACL graft should be carefully applied on ST during fixation to avoid stress shielding of the graft.

## Introduction

Anterior cruciate ligament (ACL) injury is a common sports-related knee trauma with steadily increasing incidence over recent years, exceeding seven injuries per 100,000 games among adolescent athletes (Bram et al., 2021). Due to the critical role of an intact ACL in maintaining knee kinematics (Markolf et al., 2009), ligament reconstruction has long time been the gold standard treatment for ACL injury in restoring stability, preventing early degeneration, and achieving return to preinjury sports (Lai et al., 2018). Despite the well-accepted clinical outcomes of arthroscopic ACL reconstruction (ACLR) using soft tissue grafts, the long process of ligamentization could make the grafts vulnerable to reinjury (Claes et al., 2011), and a high risk of graft failure would be worrying (Samuelsen et al., 2017). Previous studies demonstrated that outcomes after ACLR was related to graft choices (Mouarbes et al., 2019). Considering the inherent biological characteristics of commonly used grafts, interest in methods to increase the strength of graft construct has gradually risen in past decades.

Although synthetic devices to replace the ACL graft have been available since the 1970s, high failure rates and complications such as effusion and synovitis have been reported and limited their wide use (Batty et al., 2015). Other than total substitution, an addition of a broad, braided, ultrahigh-molecular-weight polyethylene/polyester suture tape (ST) is frequently applied in the repairing surgery for soft tissue, including at least the rotator cuff tendon (Boksh et al., 2022), ulnar collateral ligament (Carr et al., 2020), and lateral ankle ligament (Lewis et al., 2021). For the ACL injury, adding an ST as an internal brace was reported to improve the clinical outcomes of ACL suture repair, with comparable results in subjective scores, knee stability, and graft failure rate to standard ACLR (Murray et al., 2020; Hoogeslag et al., 2022). Such inspiring findings indicate that adding an ST to the ACL graft might promote graft healing and remodeling and probably reduce the residual laxity and graft rupture for patients undergoing ACLR.

**FIGURE 1:**
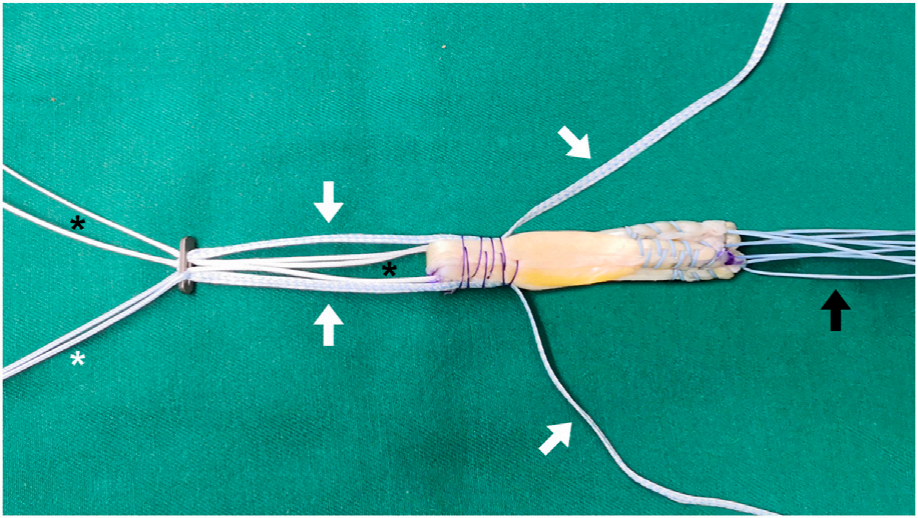
The final construct of ST-augmented quadrupled (8-strand) semitendinosus and gracilis tendon autograft, with the ST passing through the femoral button eyelets. White arrow: ST; black arrow: braided nonabsorbable suture; white asterisk: pull suture; black asterisk: adjustable loop suture. ST, suture tape.

Currently, the ST augmentation (STA) technique has been described as a novel way to enhance the graft for ACLR (Figure 1), supporting the graft in a load-sharing manner and acting as a “safety belt” to prevent the graft from excessive tension, especially in the early phase of ligamentization (Bachmaier et al., 2018). This effect was supported by biomechanical studies demonstrating that ACLR with STA improved graft stiffness and failure load compared to ACLR alone, reducing tibial displacement under anterior load (Torres et al., 2022). In the clinical scenario, the STA technique has been used for ACLR in several sports medicine centers worldwide in recent 5 years (Mackenzie et al., 2022). However, the clinical superiority of ST-augmented grafts remained unclear, and a summary of available literature on clinical outcomes of ACLR with STA is lacking.

Therefore, the purpose of this study was to systematically 1) summarize the specific technique of STA and 2) evaluate the clinical outcomes after ACLR with STA. It was hypothesized that ACLR using ST-augmented grafts would be associated with better functional outcome measures and lower graft failure rates compared to standard ACLR.

## Methods

This systematic review was performed following the Preferred Reporting Items for Systematic Reviews and Meta-Analyses (PRISMA) 2020 statement (Page et al., 2021) and was registered in INPLASY (registration number: INPLASY2022100125).

### Search strategy

An electronic search of PubMed and Embase databases was conducted on 12 September 2022 to explore studies reporting clinical outcomes of ACLR using ST-augmented grafts. The following key terms were used for the electronic search: (*anterior cruciate ligament* OR *ACL*) AND (*tape* OR *augment* OR *reinforce* OR *internal brace*). The full search strategies used for each of these electronic databases are available in Supplementary Appendix Tables S1, S2. A manual search of Google Scholar was then performed to identify studies not indexed by the Web of Science.

Two authors (Z.Z. and Z.F.) independently assessed all studies and cross-checked the finalized ones according to the inclusion and exclusion criteria (Table 1). The titles and abstracts were initially screened for relevance, then the full texts were critically retrieved for further selection. Reference lists of all included articles were reviewed for potentially eligible studies. Any disagreement about a study’s inclusion was resolved by discussion with a third senior author (H.Z.) involved if consensus could not be achieved.

**TABLE 1:** Study inclusion and exclusion criteria.

(1) Studies reporting clinical outcomes of suture tape-augmented auto- or allografts for ACL reconstructions	(1) Studies unrelated to the suture tape
	(2) Studies with the suture tape used for partial ACL injuries
(2) Studies with an adequate description of the construct of augmented grafts	(3) Studies with the suture tape used for ACL repairs
(3) Level of evidence, 1–4	(4) Studies with the suture tape used for other knee ligaments
(4) English-language articles	
(5) Studies without limits placed on the date of publication	(5) Studies with other artificial synthetic devices used for ACL reconstructions
(6) Studies published online or in print in a peer-reviewed journal	(6) Biomechanical studies, reviews, case reports, or technical notes

ACL, anterior cruciate ligament.

### Data extraction

Each finally included study was abstracted regarding the study features, patient data, surgical information, and outcome measures. Two authors (G.S. and Y.L.) independently extracted the original data, and the final decision on the disagreement was made by a third senior author (H.Z.).

Study features consisted of author name, publication year, journal, study design, level of evidence, and methodological quality. Patient data comprised number of cases, sex, age, length of follow-up, and meniscal status. Surgical information was extracted from the specific descriptions in the original studies, including graft details (choice, construct, diameter, and fixation), tape details (indication, product, and fixation), and concomitant procedures. For outcome measures, all subjective and objective results including pain and function scores, knee laxity measurements, return to sports, graft failures, and other complications were documented. Comparison analysis between pre- and postoperative conditions and between patients with and without STA was recorded. Radiographic and arthroscopic outcomes extracted by follow-up magnetic resonance imaging (MRI) and second-look arthroscopy were not reviewed in this systematic review, as there was no available data in the original studies.

Descriptive statistics were used to report study characteristics, patient data, surgical information, and outcome measures. A quantitative comparison (meta-analysis) of the data was considered inappropriate due to the heterogeneity between studies.

### Quality appraisal

The methodologic quality of each included study was assessed with the Methodological Index for Non-Randomized Studies (MINORS). The items in the MINORS criteria for non-randomized studies were scored as 0 (not reported), 1 (reported but inadequate), or 2 (reported and adequate) (Slim et al., 2003). For comparative studies, the ideal MINORS score was 24, and a study was considered at low risk of bias when it scored 21–23 and at high risk of bias when it scored ≤20. For non-comparative studies, corresponding thresholds were 16, 13–15, and ≤12 (Slim et al., 2003). The MINORS score of each study was calculated independently by two authors (G.S. and Y.L.). Any disagreement was resolved by discussion until a consensus was reached.

## Results

### Literature search

**FIGURE 2:**
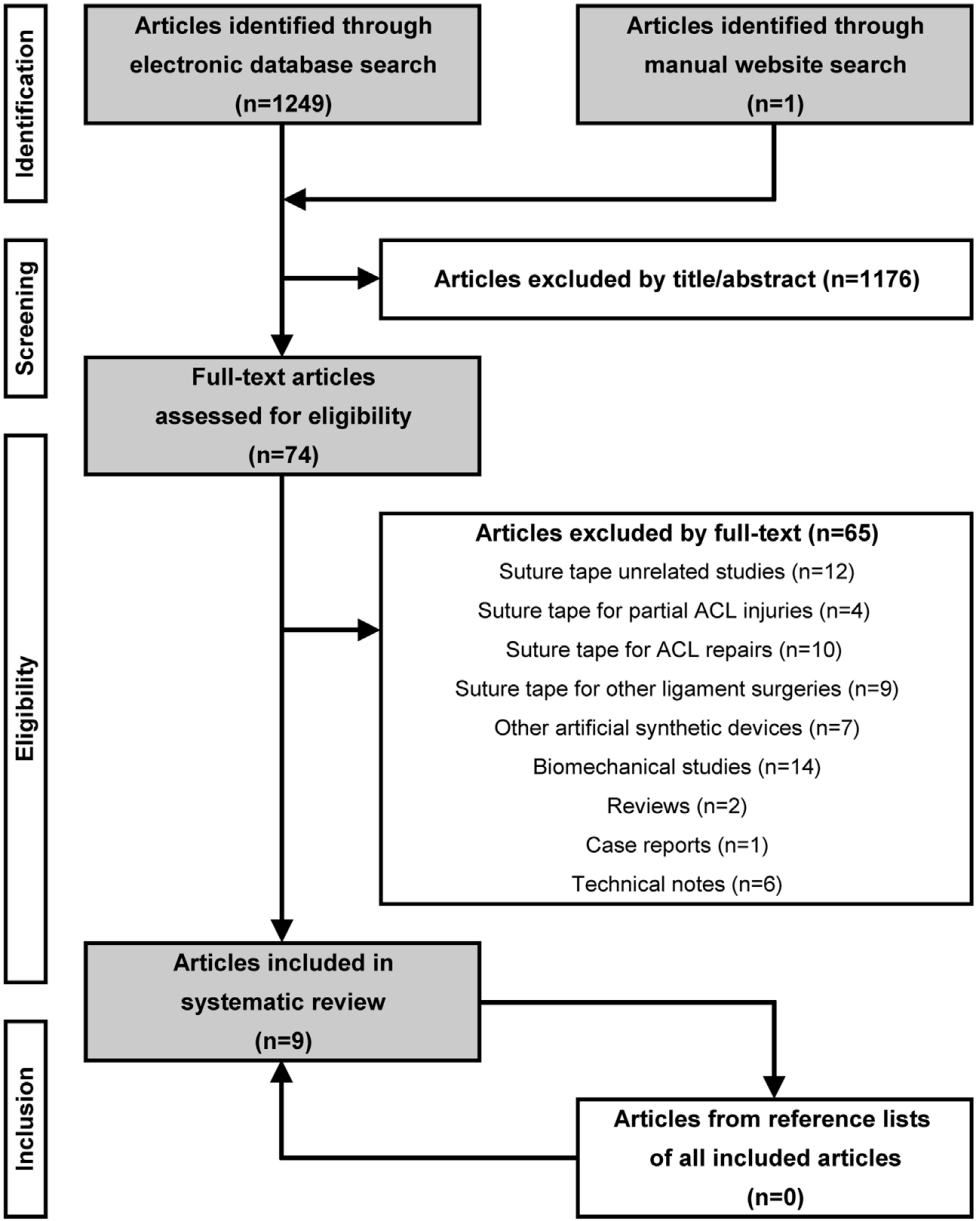
Flowchart of search strategy following the PRISMA 2020 statement (Page et al., 2021). ACL, anterior cruciate ligament.

The electronic and manual search identified 1,250 studies for initial screening, and 74 studies proceeded to full-text review. Critical application of the inclusion and exclusion criteria finally yielded nine studies (Figure 2). One was a prospective cohort study (level 2) (Shantanu et al., 2019), six were retrospective cohort studies (level 3) (Bodendorfer et al., 2019; Parkes et al., 2021; Allom et al., 2022; Kitchen et al., 2022; Szakiel et al., 2022; von Essen et al., 2022), and two were case series (level 4) (Lavender et al., 2021; Duong et al., 2022). All seven comparative studies were designed to compare patients who underwent ACLR with and without ST-augmented grafts (Table 2).

**TABLE 2:** Study feature and methodologic quality.

First author	Year	Journal	Design	LOE	MINORS	Subjective score	Laxity	RTS	Failure
Shantanu	2019	Int J Orthop Sci	Pro cohort	2	16/24	Lysholm	Yes	No	Yes
Allom	2022	Arthrosc Sports Med Rehabil	Retro cohort	3	16/24	No	Yes	No	Yes
Kitchen	2022	Orthop J Sports Med	Retro cohort	3	17/24	Lysholm, SANE, Tegner, VAS	No	Yes	No
von Essen	2022	J Exp Orthop	Retro cohort	3	19/24	KOOS	Yes	No	Yes
Parkes	2021	Arthroscopy	Retro cohort	3	20/24	Lysholm, IKDC, Tegner	Yes	No	Yes
Bodendorfer	2019	Arthroscopy	Retro cohort	3	20/24	IKDC, KOOS, SANE, VAS, WOMAC	No	Yes	No
Szakiel	2022	J Orthop	Retro cohort	3	18/24	KOOS	No	No	No
Lavender	2021	Arthrosc Sports Med Rehabil	Case series	4	11/16	IKDC	Yes	Yes	Yes
Duong	2022	Asia Pac J Sports Med Arthrosc Rehabil Technol	Case series	4	11/16	Lysholm, IKDC (grade)	Yes	No	Yes

LOE, level of evidence; MINORS, Methodological Index for Non-Randomized Studies; RTS, return to sports; pro, prospective; retro, retrospective; IKDC, international knee documentation committee; KOOS, knee injury and osteoarthritis outcome score; SANE, single assessment numeric evaluation; VAS, visual analogue scale; WOMAC, Western Ontario and McMaster Universities Osteoarthritis Index.

### Quality appraisal

A high risk of bias was confirmed in all seven comparative studies with a mean MINORS value of 18.0 ± 1.6 (range, 16–20). The two non-comparative studies were also at high risk of bias with a MINORS value of 11 for each (Table 2). The risk of bias mainly came from an unblinded assessment of the study endpoint, followed by a lack of prospective calculation of the study size and a historical comparison between the study and control group. The full appraisal for each study is available in Supplementary Appendix Table S3.

### Patient characteristics

A total of 314 knees of 314 patients undergoing ACLR with STA were enrolled in this systematic review (Table 3). There were 190 (60.5%) males and 124 females (39.5%) with a mean age of 26.6 years (range, 15.7–33.0 years). The mean clinical follow-up was 16.7 months (range, 6.0–29.0 months). The meniscal status at the time of ACLR was documented in six studies, and the incidence of meniscal tears was 49.0% (125/255) overall, 32.0% (72/225) for the medial, and 32.9% (74/225) for the lateral.

**TABLE 3:** Patient characteristics between anterior cruciate ligament reconstruction with or without suture tape augmentation.

First author	Size		Sex (M/F)		Age (y)		F-U (mo)		Meniscal tear		MM tear		LM tear	
	Tape	No tape	Tape	No tape	Tape	No tape	Tape	No tape	Tape	No tape	Tape	No tape	Tape	No tape
Shantanu	25	25	21/4	20/5*	27.8	32.2*	6.0	6.0*	NR	NR	NR	NR	NR	NR
Allom	72	97	41/31	53/44*	25.6	27.6*	6.0	6.0*	22	46*	11	29*	11	17*
Kitchen	40	40	19/21	18/22*	15.7	14.9*	27.6	29.0*	19	27*	9	7*	23	15*
von Essen	40	40	23/17	23/17*	29.2	29.2*	12.0	12.0*	23	20*	14	10*	9	10*
Parkes	36	72	25/11	50/22*	25.3	24.9*	26.1	31.3*	27	48*	18	48*	21	48*
Bodendorfer	30	30	13/17	13/17*	29.3	29.7*	29.0	30.1*	8	10*	NR	NR	NR	NR
Szakiel	23	23	12/11	11/12*	31.5	31.5*	24.0	24.0*	NR	NR	NR	NR	NR	NR
Lavender	11	NN	8/3	NN	25.5	NN	24.0	NN	NR	NN	NR	NN	NR	NN
Duong	37	NN	28/9	NN	33.0	NN	12.0	NN	26	NN	20	NN	10	NN
Overall	314	327	190/124	188/139	26.6	26.5	16.7	18.6	125	151	72	94	74	90

M, male; F, female; F-U, follow-up; MM, medial meniscus; LM, lateral meniscus; NR, not reported; NN, not needed.

*no significant difference (*p* > 0.05) between grafts with or without suture tape.

### Surgical information

ACLRs were performed with 283 hamstring tendon auto- or allografts in eight studies, 20 quadriceps tendon autografts in one study, and 11 bone-patellar tendon-bone autografts in one study (Table 4). ACL grafts were augmented with FiberTape (Arthrex, United States) in 231 patients and InternalBrace (Arthrex, United States) in 11 patients, and the product used in the rest 72 patients was not described. Different fixation methods of the graft-ST construct were applied. On the femoral side, the suspensory device was used in eight studies, with the ST passing through the eyelets of the suspensory button in eight studies and the loop of the suspensory button in two studies. On the tibial side, the suspensory device was used in seven studies and the interference screw in two studies, with the ST fixed independently from the graft using an additional anchor before graft fixation in six studies and along with the graft using the same fixation device in two studies. Most studies applied an equal or slightly less tension on the ST than the ACL graft during fixation. For all studies, the use of ST was based on the surgeon’s preference, except for one prospective cohort study with randomized selection. Concomitant lateral extra-articular tenodesis was performed in one study (Allom et al., 2022) for 84/169 patients with risk factors for graft rupture.

**TABLE 4:** Surgical technique of anterior cruciate ligament reconstruction with suture tape augmentation.

First author	Size	Graft					Suture tape		
		Choice	Strand	Diameter, mm	Fem fix	Tib fix	Product	Fem fix	Tib fix
Shantanu	25	HT (ST + G)	Quadrupled	NR	SD	NR	FiberTape	Loop	NR
Allom	72	HT (ST ± G)	Quadrupled	≥8.5 (M), ≥8 (F)	SD	SD	NR	Eyelets/loop	Add anchor
Kitchen	40	HT (ST + G)	Quadrupled	<8 (29), ≥8 (11)	SD	IS	FiberTape	Eyelets	With graft
von Essen	20	HT (ST)	Quadrupled	8–10	SD	SD	FiberTape	Eyelets	With graft
	20	QT	Quad	10 (width)	SD	SD	FiberTape	Eyelets	With graft
Parkes	36	HT (ST ± G)	Quadrupled	NR	SD	SD	FiberTape	Eyelets	Add anchor
Bodendorfer	30	HT (ST, auto ± allo)	Quadrupled	8–10	SD	SD	FiberTape	Eyelets	Add anchor
Szakiel	23	HT (ST)	Quadrupled	NR	SD	SD	FiberTape	Eyelets	Add anchor
Lavender	11	BPTB (auto/allo)	Quad	NR	SD	SD	InternalBrace	Eyelets	Add anchor
Duong	37	HT (ST)	Quadrupled	7–8	SD	SD	FiberTape	Eyelets	Add anchor

fem, femoral; tib, tibial; fix, fixation; HT, hamstring tendon; ST, semitendinosus tendon; G, gracilis tendon; QT, quadriceps tendon; BPTB, bone-patellar tendon-bone; auto, autograft; allo, allograft; M, male; F, female; SD, suspensory device; IS, interference screw; loop, suture tape passed through the loop of the femoral button; eyelets, suture tape passed through the eyelets of the femoral button; add, additional, NR, not reported.

### Subjective scores

Overall, eight subjective scores were reported in eight out of nine studies: the Lysholm score in four studies, International Knee Documentation Committee (IKDC) score in three studies, Knee injury and Osteoarthritis Outcome Score (KOOS) score in three studies, Single Assessment Numeric Evaluation (SANE) score in two studies, Tegner activity score in two studies, visual analogue scale (VAS) score in two studies, Western Ontario and McMaster Universities Osteoarthritis (WOMAC) score in one study, and IKDC grade in one study. The remaining one study (Allom et al., 2022) not reporting any subjective score (Table 2).

Seven studies compared subjective scores between pre- and postoperative status for ACLR with STA, and significant improvement was reported in six studies. Those used at least in two studies included the Lysholm score from 61.2 to 91.2 (Shantanu et al., 2019; Duong et al., 2022), IKDC score from 30.7 to 85.8 (Bodendorfer et al., 2019; Lavender et al., 2021), and KOOS score from 60.9 to 89.1 (Bodendorfer et al., 2019; Szakiel et al., 2022; von Essen et al., 2022). The IKDC grade was reported in one study (Duong et al., 2022) with the grade (A/B/C/D) from 0/0/19/18 to 30/7/0/0. The comparison of the other three scores was only performed in one study (Bodendorfer et al., 2019) with the SANE score from 96.0 to 90.0, VAS score from 7.1 to 1.6, and WOMAC score from 35.5 to 2.2. Only one study (Parkes et al., 2021) demonstrated no significant improvement in the Tegner score (from 7.2 to 7.1).

Six studies compared subjective scores between ACLR with or without STA at the final follow-up. The Tegner score used in two studies (Parkes et al., 2021; Kitchen et al., 2022) both yielded superior results in the augmentation group. Results of other scores were inconsistent across studies. Only one study (Bodendorfer et al., 2019) reported that the VAS (1.6 *versus* 3.4), IKDC (87.6 *versus* 73.2), KOOS (92.2 *versus* 87.1), SANE (90.0 *versus* 80.0), and WOMAC (2.2 *versus* 6.2) score were all significantly better in augmentation group. However, no significant superiority of any score other than the Tenger was found in the augmentation group among other studies (Table 5).

**TABLE 5:** Postoperative subjective scores between anterior cruciate ligament reconstruction with or without suture tape augmentation.

First author	Graft choice	Size		Mean VAS		Mean Lysholm		Mean IKDC		Mean KOOS		Mean SANE		Mean Tegner	
		Tape	No tape	Tape	No tape	Tape	No tape	Tape	No tape	Tape	No tape	Tape	No tape	Tape	No tape
Shantanu	HT	25	25	NR	NR	87.0	87.0**	NR	NR	NR	NR	NR	NR	NR	NR
Kitchen	HT	40	40	1.0	1.0**	92.7	92.1**	NR	NR	NR	NR	90.6	87.6**	7.4	6.3*
von Essen	HT	14	19	NR	NR	NR	NR	NR	NR	83.9	88.4**	NR	NR	NR	NR
	QT	15	9	NR	NR	NR	NR	NR	NR	87.2	77.0**	NR	NR	NR	NR
Parkes	HT	36	72	NR	NR	95.6	94.0**	94.4	93.8**	NR	NR	NR	NR	7.1	6.4*
Bodendorfer	HT	30	30	1.6	3.4*	NR	NR	87.6	73.2*	92.2	87.1*	90.0	80.0*	NR	NR
Szakiel	HT	23	22	NR	NR	NR	NR	NR	NR	92.1	87.5**	NR	NR	NR	NR
Overall		183	217	1.3	2.0	92.3	92.2	91.3	87.7	89.8	86.4	90.3	84.3	7.3	6.4

VAS, visual analogue scale; IKDC, international knee documentation committee; KOOS, knee injury and osteoarthritis outcome score; SANE, single assessment numeric evaluation; HT, hamstring tendon; QT, quadriceps tendon; NR, not reported; NN, not needed.

*, significant difference (*p* < 0.05) between grafts with or without suture tape.

**, no significant difference (*p* > 0.05) between grafts with or without suture tape.

### Objective knee laxity

Knee laxity was evaluated by the Lachman test in five studies, pivot shift test in four studies, and KT-1000/2000 arthrometer test in two studies (Table 6). In all these studies, significant improvement in objective stability was reported in patients with ST-augmented grafts at the final follow-up. The comparative analysis of knee laxity between ACLR using ST-augmented grafts and not was performed in four studies. No studies reported superior objective stability in the augmentation group at the final follow-up, although one of which (Shantanu et al., 2019) reported better improvement in grades of the Lachman test as the preoperative laxity in the augmentation group was severer.

**TABLE 6:** Postoperative objective knee laxity between anterior cruciate ligament reconstruction with or without suture tape augmentation.

First author	Graft choice	Minimum F-U, mo	Size		KT-1000/2000 SSD, mm		Lachman, nor/abnor		Pivot shift, nor/abnor	
			Tape	No tape	Tape	No tape	Tape	No tape	Tape	No tape
Shantanu	HT	6	25	25	NR	NR	23/2**	21/4***	NR	NR
Allom	HT	6	72	97	1.2**	1.3***	NR	NR	NR	NR
von Essen	HT	6	20 (16*)	20 (20*)	1.4**	2.3***	16/4**	17/3***	20/0**	20/0***
	QT	6	20 (16*)	20 (12*)	1.8**	2.0***	17/3**	17/3***	20/0**	20/0***
Parkes	HT	24	35	68	NR	NR	34/1**	65/3***	35/0**	66/2***
Lavender	BPTB	6	11	NN	NR	NN	11/0**	NN	11/0**	NN
Duong	HT	12	37	NN	NR	NN	37/0**	NN	37/0**	NN
Overall			220	230	1.3	1.5	138/10	120/13	123/0	106/2

F-U, follow-up; SSD, side-to-side difference; nor, normal; abnor, abnormal; HT, hamstring tendon; QT, quadriceps tendon; BPTB, bone-patellar tendon-bone; NR, not reported; NN, not needed.

*, number of patients who underwent KT-1000/2000 arthrometer test.

**, significant difference (*p* < 0.05) between pre- and postoperative status for grafts with suture tape.

***, no significant difference (*p* > 0.05) between grafts with or without suture tape.

### Return to sports

The ability to return to preinjury sports level was evaluated in three studies, with an overall rate of 73.8% (range, 69.2%–81.8%) in patients with ST-augmented ACL grafts (Table 7). The ability to return to sports between patients with or without STA was compared in two studies. Both demonstrated no (but a trend toward) significant difference in the rate of return to sports at a minimum 2-year follow-up, and one of them (Bodendorfer et al., 2019) reported that the use of ST was significantly correlated with earlier time to return (9.2 months *versus* 12.9 months).

**TABLE 7:** Return to preinjury sports level between anterior cruciate ligament reconstruction with or without suture tape augmentation.

First author	Graft choice	Minimum follow-up	Size		Able to return		
			Tape	No tape	Tape, n (%)	No tape, n (%)	value
Kitchen	HT	24 months	39	33	27 (69.2)	17 (51.5)	*p* = 0.124
Bodendorfer	HT	24 months	30	30	23 (76.7)	17 (56.7)	*p* = 0.100
Lavender	BPTB	24 months	11	Not needed	9 (81.8)	Not needed	Not needed
Overall			80	63	59 (73.8)	34 (54.0)	

HT, hamstring tendon; BPTB, bone-patellar tendon-bone.

### Graft failures and other complications

The graft failure and other major complications were recorded in seven studies (Table 8). The overall failure rate of ST-augmented grafts was 2.3% (range, 0%–6.7%) among 266 patients. The comparison of failure between grafts with or without ST proceeded in five studies. Almost all studies reported a lower failure rate in ST-augmented grafts, but the difference did not meet statistical significance. Other complications including further meniscal tear, arthrofibrosis, cyclops lesion, and infection were also not significantly different in any of these studies.

**TABLE 8:** Graft failure and other complications between anterior cruciate ligament reconstruction with or without suture tape augmentation.

First author	Graft choice	Minimum F-U, mo	Size		Graft failure, n (%)		Other complications	
			Tape	No tape	Tape	No tape	Tape	No tape
Allom	HT	6	72	97	0 (0.0)	0 (0.0)*	1 meniscal tear	1 cyclops lesion
Kitchen	HT	24	40	40	2 (5.0)	7 (17.5)*	2 meniscal tears, 1 infection	2 meniscal tears
Von Essen	HT	24	20	20	1 (5.0)	0 (0.0)*	1 meniscal tear, 1 cyclops lesion	2 meniscal tears, 2 cyclops lesions
	QT	24	20	20	0 (0.0)	2 (10.0)*	None	2 meniscal tears, 1 cyclops lesion
Parkes	HT	24	36	72	1 (2.8)	4 (5.6)*	2 meniscal tears, 2 arthrofibrosis, 1 cyclops lesion	3 meniscal tears, 3 arthrofibrosis, 1 infection
Bodendorfer	HT	24	30	30	2 (6.7)	2 (6.7)*	2 arthrofibrosis	1 arthrofibrosis
Lavender	BPTB	24	11		0 (0.0)	NN	1 arthrofibrosis	Not needed
Duong	HT	12	37		0 (0.0)	NN	None	Not needed
Overall			266	279	6 (2.3)	15 (5.4)		

F-U, follow-up; HT, hamstring tendon; QT, quadriceps tendon; BPTB, bone-patellar tendon-bone; NN, not needed.

*, no significant difference (*p* > 0.05) between grafts with or without suture tape.

## Discussion

The most important findings of this systematic review could be summarized as follows: 1) patients with ST-augmented grafts obtained overall favorable clinical outcomes after ACLR; 2) the use of ST was associated with significantly higher Tegner activity score and a trend toward significantly better ability to return to sports compared with standard ACLR; 3) no significant differences in most subjective scores, knee laxity, and graft failure were found between patients undergoing ACLR with STA and ACLR alone; and 4) most authors preferred an equal or slightly less tension on the ST than the ACL graft during fixation.

In recent 2–5 years, the use of ST to improve the biomechanical performance of ACL graft has been a hot topic in the field of sports medicine, intending to protect the graft from over-loads during the early rehabilitation phase when the graft is weak secondary to necrosis and remodeling process during this time (Claes et al., 2011). As suggested by animal models, adding an ST improved the mechanical properties of the ACL graft by the load-sharing effect, characterized by reduced graft elongation and increased load to failure (Bachmaier et al., 2018), and this function was especially dramatic in smaller -diameter grafts (Noonan et al., 2020), occurring earlier and carrying final loads with a greater extent (Bachmaier et al., 2022). Cadaveric studies further demonstrated that the increased graft-ST construct stiffness was associated with improved knee stability after ACLR, manifested as decreased tibial displacement under anterior load when the ACL graft was augmented with ST (Torres et al., 2022).

Despite increased mechanical strength, an over-tensioned ST possibly led to stress shielding of the ACL graft, compromising the graft ligamentization process (Muellner et al., 2001). Therefore, proper tension of the ST should be carefully applied during fixation in clinical practice to avoid stress shielding of the graft. Among the included studies, the majority preferred to fix the ST independent from the graft on the tibial side, with equal or slightly less manual tension on the ST. If fixed together, the ST was pulled to just remove the slack but without applying tension on the ST. For the same purpose, some suggested fixing the ST in full or hyperextension and the graft in 30° of flexion. As proposed by biomechanical studies, an ideal augmentation effect should be dynamic, permitting lower loads transferred by the graft and only acting as a “safety belt” when strains exceed (Bachmaier et al., 2018). However, whether mechanically improved graft constructs would achieve better surgical outcomes of ACLR in clinical scenarios needs to be further verified.

For the functional outcomes in patients with ACLR and STA, this systematic review showed consistent improvements in nearly all subjective scores at a mean follow-up of 16.7 months, indicating amelioration of clinical symptoms. Meanwhile, significant improvement in objective knee stability was reported in five studies, and no positive pivot shift was observed at the final follow-up. However, as opposed to the study hypothesis, the addition of ST failed to show superiority in either subjective scores or objective stability compared to standard ACLR among nearly all included studies. Based on this evidence, improved mechanical behavior of ST-augmented grafts would not necessarily lead to better subjective function and mechanical stability after ACLR, and the clinical implication of STA requires assessments from specific aspects.

Interestingly, the only subjective score exhibiting superiority in STA was the Tegner activity score, implying better maintenance of sports level in patients using augmented grafts. Such findings were consistent with the results on the ability to return to sports that patients with STA had a significantly earlier time to return to preinjury level and a trend toward a significantly higher proportion to return to preinjury level. Meanwhile, the rate (69.2%–81.8%) of return to preinjury level in patients undergoing ACLR and STA was comparable with the literature. In a recent systematic review of ACLR in the athletic population, the rate of return to preinjury level was 32%–64% in bone-patellar tendon-bone autografts and 9%–65% in hamstring tendon autografts (DeFazio et al., 2020). The causes of better sports performance in patients with STA was unable to be clarified according to the current data since the objective stability was similar and the rehabilitation protocol was identical between patient with or without STA. A potential explanation could be the better confidence in return to sports in patients with STA as they were aware of the use of ST (Keays et al., 2022).

One of the expectations for the use of STA is to protect the ACL graft from a high risk of failure. In this systematic review, the failure rate of ST-augmented ranged from 0% to 6.7%, considered satisfactory and compared similarly to the results of previous studies that 0%–6.1% of athletes experienced graft ruptures at a minimum 2-year follow-up after standard ACLR (DeFazio et al., 2020). Nevertheless, although the use of ST was seemingly associated with a lower failure rate, the difference between grafts with or without ST was not of statistical significance. Considering that half to three-quarters of graft failures would occur in the first 1–2 years following ACLR (Webster and Feller, 2016), the mechanical protection of ST to the ACL graft might not reduce the risk of failures.

The biocompatibility of synthetic material is another concern about the intra-articular use of ST. Even for the more recent designs of synthetic ligament devices, the chronic inflammatory response of the synovium was still reported to be a common finding after ACLR using the Ligament Augmentation and Reconstruction System, which comprises fibers of terephthalic polyethylene polyester (Tulloch et al., 2019). However, histologic assessments in an animal model did not confirm any ST-associated immune responses or cartilage erosions at 6 months following ACLR and STA (Smith et al., 2019). In this systematic review, complications including arthrofibrosis, cyclops lesion, and infection were at a low incidence and similar between ACLR with or without STA, supporting the safety of ST added in ACL grafts.

Clinically, the signal intensity on MRI has been commonly used to monitor the graft ligamentization process after ACLR, with a higher intensity revealing a poor structural strength (Fleming et al., 2011). According to radiological studies, the signal intensity of ACL graft reached its peak at 6 months, followed by a gradual decline until 2–3 years postoperatively (Lansdown et al., 2020; Warth et al., 2020). Unfortunately, no included studies conducted an MRI analysis of ACL grafts. A serial evaluation of the graft signal is necessary to explore if the ligamentization process would be improved with the STA technique in future studies.

### Limitations

The limitations of this systematic review were largely from the results of input literature. Firstly, the low level of evidence and high risk of bias within the majority of included studies lowered the validity of this review. Second, the heterogeneity and insufficient data across the original literature restricted the application of a formal meta-analysis. Third, variable reports of outcome measures made this review unable to draw a firm conclusion on the superiority of STA from the clinical perspective. Fourth, the surgical indication of STA lacked a specific description, leading to uncertainty that patients with what features would more benefit from the ST-augmented grafts. Fifth, no radiological or arthroscopic methods were used to evaluate the intra-articular condition after ACLR with STA. Despite the limitations, this is the first study to systematically review the clinical outcomes of ACLR with STA, with a comparison to standard ACLR. This study could be helpful to direct future studies that seek to explore the clinical effectiveness of STA in ACLR.

## Conclusion

Clinically, the application of STA achieved overall favorable outcomes in patients with ACLR. ACLR using ST-augmented grafts was seemingly associated with better sports performance compared to standard ACLR. However, despite inconsistent reports across original studies, ACLR with STA did not yield superior results in most functional scores, knee stability measures, and graft failure rates than ACLR alone. Technically, proper tension of the ST should be carefully applied during fixation with an equal or slightly less tension on the ST than the ACL graft to avoid stress shielding of the graft.

## Data Availability

The data analyzed in this study is subject to the following licenses/restrictions: This is a systematic review using data extracted from previously published literature. Requests to access these datasets should be directed to TZ, zhengtong2020@pku.edu.cn.

## References

[B1] Allom R. J. Wood J. A. Chen D. B. Macdessi S. J. (2022). The addition of suture tape to the hamstring graft construct does not reduce instrumented knee laxity following ACL reconstruction. Arthrosc. Sports Med. Rehabilitation 4 (2), e545–e551. 10.1016/j.asmr.2021.11.015 PMC9042745 35494286

[B2] Bachmaier S. Smith P. A. Argintar E. H. Chahla J. Higgins L. D. Wijdicks C. A. (2022). Independent suture augmentation with all-inside anterior cruciate ligament reconstruction reduces peak loads on soft-tissue graft. A biomechanical full-construct study. Arthrosc. J. Arthrosc. Relat. Surg. 38 (1), 88–98. 10.1016/j.arthro.2021.09.032 34655766

[B3] Bachmaier S. Smith P. A. Bley J. Wijdicks C. A. (2018). Independent suture tape reinforcement of small and standard diameter grafts for anterior cruciate ligament reconstruction: A biomechanical full construct model. Arthrosc. J. Arthrosc. Relat. Surg. 34 (2), 490–499. 10.1016/j.arthro.2017.10.037 29275984

[B4] Batty L. M. Norsworthy C. J. Lash N. J. Wasiak J. Richmond A. K. Feller J. A. (2015). Synthetic devices for reconstructive surgery of the cruciate ligaments: A systematic review. Arthrosc. J. Arthrosc. Relat. Surg. 31 (5), 957–968. 10.1016/j.arthro.2014.11.032 25620500

[B5] Bodendorfer B. M. Michaelson E. M. Shu H. T. Apseloff N. A. Spratt J. D. Nolton E. C. (2019). Suture augmented versus standard anterior cruciate ligament reconstruction: A matched comparative analysis. Arthrosc. J. Arthrosc. Relat. Surg. 35 (7), 2114–2122. 10.1016/j.arthro.2019.01.054 31167738

[B6] Boksh K. Haque A. Sharma A. Divall P. Singh H. (2022). Use of suture Tapes versus conventional sutures for arthroscopic rotator cuff repairs: A systematic review and meta-analysis. Am. J. Sports Med. 50 (1), 264–272. 10.1177/0363546521998318 33740395

[B7] Bram J. T. Magee L. C. Mehta N. N. Patel N. M. Ganley T. J. (2021). Anterior cruciate ligament injury incidence in adolescent athletes: A systematic review and meta-analysis. Am. J. Sports Med. 49 (7), 1962–1972. 10.1177/0363546520959619 33090889

[B8] Carr J. B. Camp C. L. Dines J. S. (2020). Elbow ulnar collateral ligament injuries: Indications, management, and outcomes. Arthrosc. J. Arthrosc. Relat. Surg. 36 (5), 1221–1222. 10.1016/j.arthro.2020.02.022 32112818

[B9] Claes S. Verdonk P. Forsyth R. Bellemans J. (2011). The "ligamentization" process in anterior cruciate ligament reconstruction: What happens to the human graft? A systematic review of the literature. Am. J. Sports Med. 39 (11), 2476–2483. 10.1177/0363546511402662 21515806

[B10] Defazio M. W. Curry E. J. Gustin M. J. Sing D. C. Abdul-Rassoul H. Ma R. (2020). Return to sport after ACL reconstruction with a btb versus hamstring tendon autograft: A systematic review and meta-analysis. Orthop. J. Sports Med. 8 (12), 232596712096491. 10.1177/2325967120964919 PMC7745570 33403206

[B11] Duong T. D. Tran D. T. Do B. N. T. Nguyen T. T. Le S. M. Le H. H. (2022). All-inside arthroscopic anterior cruciate ligament reconstruction with internal brace ligament augmentation using semitendinosus tendon autograft: A case series. Asia-Pacific J. Sports Med. Arthrosc. Rehabilitation Technol. 29, 15–21. 10.1016/j.asmart.2022.05.002 PMC9256544 35847193

[B12] Fleming B. C. Vajapeyam S. Connolly S. A. Magarian E. M. Murray M. M. (2011). The use of magnetic resonance imaging to predict ACL graft structural properties. J. Biomechanics 44 (16), 2843–2846. 10.1016/j.jbiomech.2011.09.004 PMC3208804 21962290

[B13] Hoogeslag R. a. G. T Veld R. Brouwer R. W. De Graaff F. Verdonschot N. (2022). Huis inAcute anterior cruciate ligament rupture: Repair or reconstruction? Five-year results of a randomized controlled clinical trial. Am. J. Sports Med. 50 (7), 1779–1787. 10.1177/03635465221090527 35486517

[B14] Keays S. L. Mellifont D. B. Keays A. C. Stuelcken M. C. Lovell D. I. Sayers M. G. L. (2022). Long-term return to sports after anterior cruciate ligament injury: Reconstruction vs No reconstruction-A comparison of 2 case series. Am. J. Sports Med. 50 (4), 912–921. 10.1177/03635465211073152 35148249

[B15] Kitchen B. T. Mitchell B. C. Cognetti D. J. Siow M. Y. Howard R. Carroll A. N. (2022). Outcomes after hamstring ACL reconstruction with suture tape reinforcement in adolescent athletes. Orthop. J. Sports Med. 10 (4), 232596712210855. 10.1177/23259671221085577 PMC9003649 35425845

[B16] Lai C. C. H. Ardern C. L. Feller J. A. Webster K. E. (2018). Eighty-three per cent of elite athletes return to preinjury sport after anterior cruciate ligament reconstruction: A systematic review with meta-analysis of return to sport rates, graft rupture rates and performance outcomes. Br. J. Sports Med. 52 (2), 128–138. 10.1136/bjsports-2016-096836 28223305

[B17] Lansdown D. A. Xiao W. Zhang A. L. Allen C. R. Feeley B. T. Li X. (2020). Quantitative imaging of anterior cruciate ligament (ACL) graft demonstrates longitudinal compositional changes and relationships with clinical outcomes at 2 years after ACL reconstruction. J. Orthop. Res. 38 (6), 1289–1295. 10.1002/jor.24572 31868948 PMC7433779

[B18] Lavender C. Singh V. Berdis G. Fravel W. Lamba C. Patel T. (2021). Anterior cruciate ligament (ACL) reconstruction augmented with bone marrow concentrate, demineralized bone matrix, autograft bone, and a suture tape (the fertilized ACL). Arthrosc. Sports Med. Rehabilitation 3 (6), e1719–e1722. 10.1016/j.asmr.2021.07.030 PMC8689152 34977625

[B19] Lewis T. L. Joseph A. Patel A. Ahluwalia R. Ray R. (2021). Modified brostrom repair with suture tape augmentation for lateral ankle instability: A systematic review. Foot Ankle Surg. 27 (3), 278–284. 10.1016/j.fas.2020.12.004 33451906

[B20] Mackenzie C. E. A. Huntington L. S. Tulloch S. (2022). Suture tape augmentation of anterior cruciate ligament reconstruction increases biomechanical stability: A scoping review of biomechanical, animal, and clinical studies. Arthrosc. J. Arthrosc. Relat. Surg. 38 (6), 2073–2089. 10.1016/j.arthro.2021.12.036 34990759

[B21] Markolf K. L. Park S. Jackson S. R. Mcallister D. R. (2009). Anterior-posterior and rotatory stability of single and double-bundle anterior cruciate ligament reconstructions. J. Bone Jt. Surgery-American Volume 91 (1), 107–118. 10.2106/JBJS.G.01215 19122085

[B22] Mouarbes D. Menetrey J. Marot V. Courtot L. Berard E. Cavaignac E. (2019). Anterior cruciate ligament reconstruction: A systematic review and meta-analysis of outcomes for quadriceps tendon autograft versus bone-patellar tendon-bone and hamstring-tendon autografts. Am. J. Sports Med. 47 (14), 3531–3540. 10.1177/0363546518825340 30790526

[B23] Muellner T. Kwasny O. Loehnert V. Mallinger R. Unfried G. Schabus R. (2001). Light and electron microscopic study of stress-shielding effects on rat patellar tendon. Archives Orthop. Trauma Surg. 121 (10), 561–565. 10.1007/s004020100281 11768636

[B24] Murray M. M. Fleming B. C. Badger G. J. Team B. T. Freiberger C. Henderson R. (2020). Bridge-enhanced anterior cruciate ligament repair is not inferior to autograft anterior cruciate ligament reconstruction at 2 Years: Results of a prospective randomized clinical trial. Am. J. Sports Med. 48 (6), 1305–1315. 10.1177/0363546520913532 32298131 PMC7227128

[B25] Noonan B. C. Bachmaier S. Wijdicks C. A. Bedi A. (2020). Independent suture tape reinforcement of tripled smaller-diameter and quadrupled grafts for anterior cruciate ligament reconstruction with tibial screw fixation: A biomechanical full construct model. Arthrosc. J. Arthrosc. Relat. Surg. 36 (2), 481–489. 10.1016/j.arthro.2019.06.036 31901386

[B26] Page M. J. Moher D. Bossuyt P. M. Boutron I. Hoffmann T. C. Mulrow C. D. (2021). PRISMA 2020 explanation and elaboration: Updated guidance and exemplars for reporting systematic reviews. BMJ 372, n160. 10.1136/bmj.n160 33781993 PMC8005925

[B27] Parkes C. W. Leland D. P. Levy B. A. Stuart M. J. Camp C. L. Saris D. B. F. (2021). Hamstring autograft anterior cruciate ligament reconstruction using an all-inside technique with and without independent suture tape reinforcement. Arthrosc. J. Arthrosc. Relat. Surg. 37 (2), 609–616. 10.1016/j.arthro.2020.09.002 PMC7867617 33144236

[B28] Samuelsen B. T. Webster K. E. Johnson N. R. Hewett T. E. Krych A. J. (2017). Hamstring autograft versus patellar tendon autograft for ACL reconstruction: Is there a difference in graft failure rate? A meta-analysis of 47, 613 patients. Clin. Orthop. Relat. Res. 475 (10), 2459–2468. 10.1007/s11999-017-5278-9 28205075 PMC5599382

[B29] Shantanu K. Singh S. Ratha S. Kumar D. Sharma V. (2019). Comparative study of functional outcomes of arthroscopic ACL reconstruction by augmented hamstring graft with fiber tape and hamstring graft alone: A prospective study. Int. J. Orthop. Sci. 5 (3), 165–173. 10.22271/ortho.2019.v5.i3d.1526

[B30] Slim K. Nini E. Forestier D. Kwiatkowski F. Panis Y. Chipponi J. (2003). Methodological index for non-randomized studies (minors): Development and validation of a new instrument. ANZ J. Surg. 73 (9), 712–716. 10.1046/j.1445-2197.2003.02748.x 12956787

[B31] Smith P. A. Bozynski C. C. Kuroki K. Henrich S. M. Wijdicks C. A. Cook J. L. (2019). Intra-articular biocompatibility of multistranded, long-chain polyethylene suture tape in a canine ACL model. J. Knee Surg. 32 (6), 525–531. 10.1055/s-0038-1655765 29852514

[B32] Szakiel P. M. Aksu N. E. Kirloskar K. M. Gruber M. D. Zittel K. W. Grieme C. V. (2022). Rehabilitation and functional outcomes in internally braced and standard ACL reconstructions. J. Orthop. 33, 95–99. 10.1016/j.jor.2022.07.002 35899098 PMC9310076

[B33] Torres S. J. Nelson T. J. Pham N. Uffmann W. Limpisvasti O. Metzger M. F. (2022). Suture tape augmentation increases the time-zero stiffness and strength of anterior cruciate ligament grafts: A cadaveric study. Arthrosc. Sports Med. Rehabilitation 4 (4), e1253–e1259. 10.1016/j.asmr.2022.02.008 PMC9402422 36033200

[B34] Tulloch S. J. Devitt B. M. Norsworthy C. J. Mow C. (2019). Synovitis following anterior cruciate ligament reconstruction using the LARS device. Knee Surg. Sports Traumatol. Arthrosc. 27 (8), 2592–2598. 10.1007/s00167-018-5280-0 30406813

[B35] Von Essen C. Sarakatsianos V. Cristiani R. Stalman A. (2022). Suture tape reinforcement of hamstring tendon graft reduces postoperative knee laxity after primary ACL reconstruction. J. Exp. Orthop. 9 (1), 20. 10.1186/s40634-022-00454-2 35195796 PMC8866616

[B36] Warth R. J. Zandiyeh P. Rao M. Gabr R. E. Tashman S. Kumaravel M. (2020). Quantitative assessment of *in vivo* human anterior cruciate ligament autograft remodeling: A 3-dimensional UTE-T2* imaging study. Am. J. Sports Med. 48 (12), 2939–2947. 10.1177/0363546520949855 32915640

[B37] Webster K. E. Feller J. A. (2016). Exploring the high reinjury rate in younger patients undergoing anterior cruciate ligament reconstruction. Am. J. Sports Med. 44 (11), 2827–2832. 10.1177/0363546516651845 27390346

