# Supplementary material for: Suture tape augmentation, a novel application of synthetic materials in anterior cruciate ligament reconstruction: A systematic review
Source: Front Bioeng Biotechnol. 2023 Jan 3;10:1065314. doi: 10.3389/fbioe.2022.1065314 (PMC9850216; doi:10.3389/fbioe.2022.1065314)
Supplement: Supplementary file 1 [file DataSheet1.docx]

**Appendix Table 1** Full Search Strategy for PubMed Database

| Search Date: September 12, 2022 |
| --- |
| 1. "anterior cruciate ligament"[MeSH Terms] |
| 2. "anterior"[All Fields] |
| 3. "cruciate"[All Fields] |
| 4. "ligament"[All Fields] |
| **5. 2 AND 3 AND 4** |
| 6. "anterior cruciate ligament"[All Fields] |
| 7. "acl"[All Fields] |
| **8. 1 OR 5 OR 6 OR 7** |
| 9. "tape"[All Fields] |
| 10. "augment"[All Fields] |
| 11. "augmentation"[All Fields] |
| 12. "augmentations"[All Fields] |
| 13. "augmented"[All Fields] |
| 14. "augmenting"[All Fields] |
| 15. "augments"[All Fields] |
| **16. 10 OR 11 OR 12 OR 13 OR 14 OR 15** |
| 17. "reinforce"[All Fields] |
| 18. "reinforced"[All Fields] |
| 19. "reinforcement"[All Fields] |
| 20. "reinforcements"[All Fields] |
| 21. "reinforcer"[All Fields] |
| 22. "reinforcer's"[All Fields] |
| 23. "reinforcers"[All Fields] |
| 24. "reinforces"[All Fields] |
| 25. "reinforcing"[All Fields] |
| **26. 17 OR 18 OR 19 OR 20 OR 21 OR 22 OR 23 OR 24 OR 25** |
| 27. "internal"[All Fields] |
| 28. "internally"[All Fields] |
| 29. "internals"[All Fields] |
| **30. 27 OR 28 OR 29** |
| 31. "brace's"[All Fields] |
| 32. "braced"[All Fields] |
| 33. "braces"[MeSH Terms] |
| 34. "braces"[All Fields] |
| 35. "brace"[All Fields] |
| 36. "bracing"[All Fields] |
| **37. 31 OR 32 OR 33 OR 34 OR 35 OR 36** |
| **38. 30 AND 37** |
| **39. 9 OR 16 OR 26 OR 38** |
| **40. 8 AND 39** |

**Appendix Table 2** Full Search Strategy for Embase Database

| Search Date: September 12, 2022 |
| --- |
| 1. 'anterior cruciate ligament'/exp |
| 2. 'anterior cruciate ligament' |
| 3. anterior |
| 4. cruciate |
| 5. 'ligament'/exp |
| 6. ligament |
| **7. 5 OR 6** |
| **8. 3 AND 4 AND 7** |
| 9. acl |
| **10. 1 OR 2 OR 8 OR 9** |
| 11. tape |
| 12. augment |
| 13. reinforce |
| 14. 'internal brace' |
| 15. internal |
| 16. 'brace'/exp |
| 17. brace |
| **18. 16 OR 17** |
| **19. 15 AND 18** |
| **20. 11 OR 12 OR 13 OR 14 OR 19** |
| **21. 10 AND 20** |

**Appendix Table 3** Methodologic Quality of Each Study Assessed with the Methodological Index for Non-Randomized Studies (MINORS)

| First Author | Year | Non-Comparative Studies | | | | | | | | Comparative Studies | | | | Total Score | Risk of Bias |
| --- | --- | --- | --- | --- | --- | --- | --- | --- | --- | --- | --- | --- | --- | --- | --- |
|  |  | C1 | C2 | C3 | C4 | C5 | C6 | C7 | C8 | C9 | C10 | C11 | C12 |  |  |
| Shantanu | 2019 | 2 | 2 | 2 | 2 | 0 | 1 | 2 | 0 | 2 | 2 | 0 | 1 | 16/24 | High |
| Allom | 2022 | 2 | 2 | 2 | 2 | 0 | 1 | 2 | 0 | 2 | 0 | 1 | 2 | 16/24 | High |
| Kitchen | 2022 | 2 | 2 | 2 | 2 | 0 | 2 | 1 | 0 | 2 | 0 | 2 | 2 | 17/24 | High |
| von Essen | 2022 | 2 | 2 | 2 | 2 | 0 | 1 | 1 | 1 | 2 | 2 | 2 | 2 | 19/24 | High |
| Parkes | 2021 | 2 | 2 | 2 | 2 | 1 | 2 | 1 | 1 | 2 | 1 | 2 | 2 | 20/24 | High |
| Bodendorfer | 2019 | 2 | 2 | 2 | 2 | 1 | 2 | 1 | 2 | 2 | 0 | 2 | 2 | 20/24 | High |
| Szakiel | 2022 | 2 | 2 | 2 | 1 | 0 | 2 | 2 | 0 | 2 | 2 | 1 | 2 | 18/24 | High |
| Lavender | 2021 | 2 | 2 | 2 | 2 | 0 | 2 | 1 | 0 | NN | NN | NN | NN | 11/16 | High |
| Duong | 2022 | 2 | 2 | 2 | 2 | 0 | 1 | 2 | 0 | NN | NN | NN | NN | 11/16 | High |

C, criterion; C1, a clearly stated aim; C2, inclusion of consecutive patients; C3, prospective collection of data; 4, endpoints appropriate to the aim of the study; C5, unbiased assessment of the study endpoint; C6, follow-up period appropriate to the aim of the study; C7, loss to follow up less than 5%; C8, prospective calculation of the study size; C9, an adequate control group; C10, contemporary groups; C11, baseline equivalence of groups; C12, adequate statistical analyses; NN, not needed.
